# Circadian and Sleep Modulation of Dreaming in Women with Major Depression

**DOI:** 10.3390/clockssleep4010012

**Published:** 2022-02-28

**Authors:** Angelina Birchler-Pedross, Sylvia Frey, Christian Cajochen, Sarah L. Chellappa

**Affiliations:** 1 Centre for Chronobiology, Psychiatric Hospital of the University of Basel, CH-4002 Basel, Switzerland; anbirchler@hotmail.com (A.B.-P.); sfrey@quickline.ch (S.F.); christian.cajochen@upk.ch (C.C.); 2 Transfaculty Research Platform Molecular and Cognitive Neurosciences, University of Basel, CH-4002 Basel, Switzerland; 3 Department of Nuclear Medicine, Faculty of Medicine and University Hospital Cologne, University of Cologne, 50937 Cologne, Germany

**Keywords:** dream, emotionality, circadian rhythms, NREM sleep, REM sleep, depression, mental health

## Abstract

Growing evidence indicates an association between reduced dream recall and depressive symptomatology. Here, we tested the prediction that reduced dream recall in individuals experiencing major depressive disorder (MDD) is due to alterations in circadian and sleep processes. Nine young healthy women (20–31 years) and eight young unmedicated women (20–31 years) diagnosed with MDD underwent a 40 h multiple nap protocol with ten alternating cycles of 150 min wake/75 min sleep under a stringently controlled circadian laboratory protocol. After each nap, we assessed dream recall, number of dreams and dream emotional load using the Sleep Mentation Questionnaire. Dream recall and the number of dreams did not significantly differ between groups (pFDR > 0.1). However, there was a significant difference for the dream emotional load (interaction of “Group” vs. “Time”, pFDR = 0.01). Women with MDD had a two-fold higher (negative) emotional load as compared to healthy control women, particularly after naps during the circadian night (between ~22:00 h and ~05:00 h; Tukey–Kramer test, *p* = 0.009). Furthermore, higher (negative) dream emotional load was associated with impaired mood levels in both groups (R^2^ = 0.71; *p* < 0.001). Our findings suggest that the circadian and sleep modulation of dreaming may remain intact in unmedicated young women experiencing MDD.

## 1. Introduction

Depression is a major public health concern that affects ~265 million people worldwide [1]. Importantly, it is one of the leading causes of disability and morbidity because its (long-lasting) effects drastically impair an individual’s ability to function and to live a satisfying life [1]. Depression includes a constellation of symptoms, such as persistent sadness, lack of interest or pleasure in previously deemed rewarding activities, sleep and appetite disruptions, and tiredness [2]. It is often associated with disrupted sleep patterns to the extent that ~50% of patients with depression show clinically diagnosed (comorbid) insomnia [3,4]. Meta-analyses show that insomnia symptoms can double the odds of developing depression [5,6], while cognitive behavioral therapy for insomnia can lower rates of depression recurrence [7,8]. Beyond disturbances in sleep–wake regulation, the circadian system is an essential modulator for affective control in healthy individuals [9,10], in individuals engaged in shift work [11,12] and in those experiencing depression [13]. Women with mild-to-moderate depression may exhibit lower mood levels over time that might reflect altered endogenous circadian mood rhythms [13]. Collectively, these data point to a critical role of circadian and sleep processes in the pathophysiology of depression.

Dreaming is a distinctive state of consciousness during sleep generated by specific neural circuits, including those encompassing the hippocampal memory system [14], whereby perceptual imagery is generated in the absence of sensory input [15,16]. Dreaming occurs predominantly during rapid eye movement (REM) sleep, such that dream recall estimates following REM sleep are ~80%, whereas it is less than 10% after non-rapid eye movement (NREM) sleep [17,18]. However, there is some evidence that dream recall can be associated with a local decrease in electroencephalographic (EEG) low-frequency activity (0.75–4 Hz) during NREM and REM sleep [19,20,21]. Hence, different sleep stages likely support dreaming. Importantly, dreaming also depends on circadian-driven changes in brain activity, particularly the circadian regulation of REM sleep [22]. The functional relevance of dreaming has been a matter of intense debate over many decades, and converging lines of research posit that dreams can aid in the emotional regulation and processing of negative affect [18,23]. Evidence suggests an association of dream recall, dream emotional load and psychological components of depression [18,24,25]. Accordingly, individuals experiencing depression likely have reduced dream recall [25,26,27], with higher negative dream emotional load [27], and the latter may be associated with increased suicidal ideation [4]. Collectively, these findings suggest that dream assessments may offer a unique window to understand disruptions of affective control in this vulnerable patient population.

**Figure 1:**
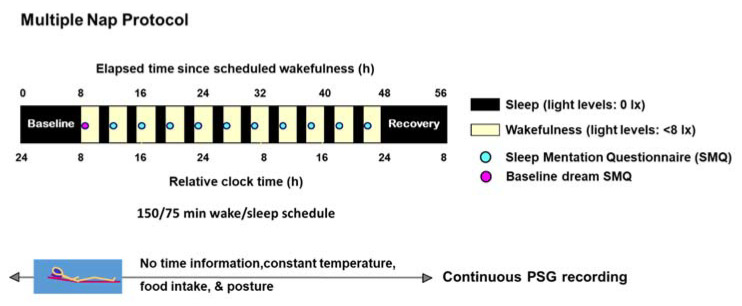
Schematic illustration of the 40 h multiple nap protocol. Overview of the timing of sleep and wakefulness and dream assessments in healthy controls and women with major depressive disorder (MDD) during the nap protocol (see “Study design, Section 4.2” for details). The illustration presented as relative clock time. Abbreviations: PSG: polysomnography.

Here, we tested the prediction that reduced dream recall in individuals experiencing depression is due to a disruption of the circadian modulation of dreaming. Moreover, we tested the prediction that reduced dream recall in depression occurs following naps with REM sleep, which is purported to be the sleep stage most conducive for dreaming [17,18] (for study protocol, see Figure 1). Lastly, we also assessed whether higher (negative) dream emotional load was associated with worse mood levels. 

## 2. Results

### 2.1. Baseline Dream Recall, Number of Dreams and Dream Emotional Load

As a first step, we assessed whether there were group differences in baseline dream recall, the number of dreams, and the dream emotional load assessed immediately after the baseline sleep episode. Accordingly, we observed no significant group differences for baseline dream recall (mean and standard error of the mean [SEM]: healthy control women: 1.2 ± 0.4, women with MDD: 1.1 ± 0.7, Mann–Whitney U test, *p* = n.s.). The baseline number of dreams did not significantly differ between groups (healthy control women: 1.7 ± 0.3, women with MDD: 1.4 ± 0.4, Mann–Whitney U test, *p* = n.s.). Likewise, we observed no significant baseline group differences for the dream emotional composite score (healthy control women: 1.4 ± 0.5, women with MDD: 1.8 ± 0.3, Mann–Whitney U test; *p* = n.s.).

### 2.2. Circadian Modulation of Dream Recall, Number of Dreams and Dream Emotional Load 

**Figure 2:**
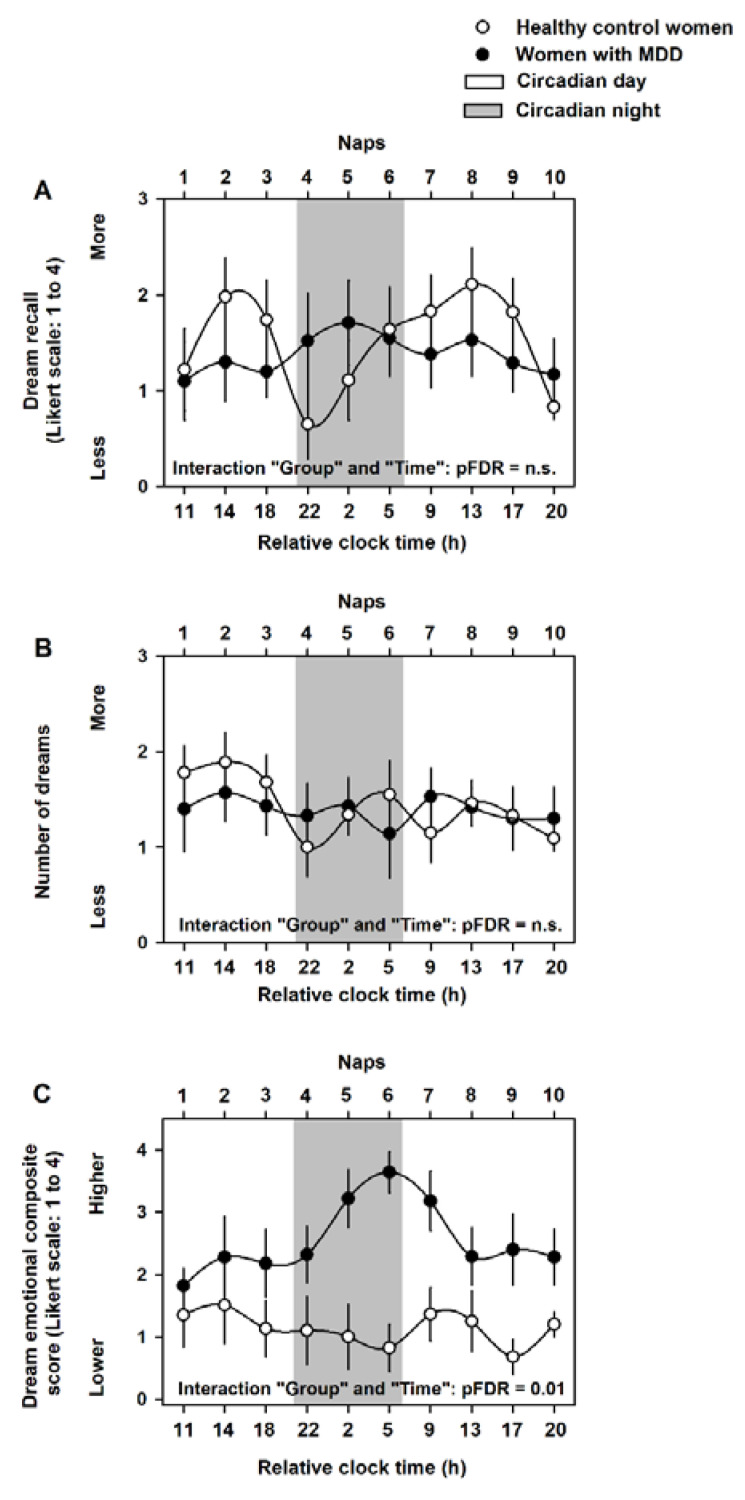
Dream recall, number of dreams and dream emotional composite score levels during the 40 h multiple nap protocol. We observed no significant interaction effects of “Group” and “Time” for dream recall (**A**) and the number of dreams (**B**). Conversely, we observed a significant interaction effect of “Group” and “Time” for the dream emotional composite score (**C**). Women with MDD showed significantly higher (negative) dream emotional score as compared to the healthy control women, particularly during the circadian night. Data correspond to the mean and standard error of the mean (SEM). Black circles are the average data for women with MDD, and white circles are the average data for the healthy control women. Grey bars correspond to the naps during the circadian night (between ~22:00 h and ~05:00 h).

Mixed-model analyses of variance of dream recall yielded significant differences for the effect of “time” (F = 7.2, pFDR < 0.001), but not for the effect of “Group” (F = 2.3, pFDR = n.s.) or for the interaction effect of “Group” and “Time” (F = 1.2, pFDR = n.s.; Figure 2A). Similarly, the analyses of number of dreams yielded significant differences for the effect of “time” (F = 4.2, pFDR < 0.001), but not for the effect of “Group” (F = 2.4, pFDR = n.s.) or for the interaction effect of “Group” and “Time” (F = 0.8, pFDR = n.s.; Figure 2B). In contrast, analyses of the dream emotional composite score yielded significant differences for the effect of “time” (F = 81, pFDR < 0.001), the effect of “Group” (F = 5.9, pFDR = 0.01) and for the interaction effect of “Group” and “Time” (F = 4.2, pFDR = 0.01). Accordingly, women with MDD had a significantly higher (negative) dream emotional score as compared to the healthy control women (healthy control women: 1.1 ± 0.5; women with MDD: 2.6 ± 0.4, Tukey-Kramer test, *p* = 0.009; Figure 2C).

**Figure 3:**
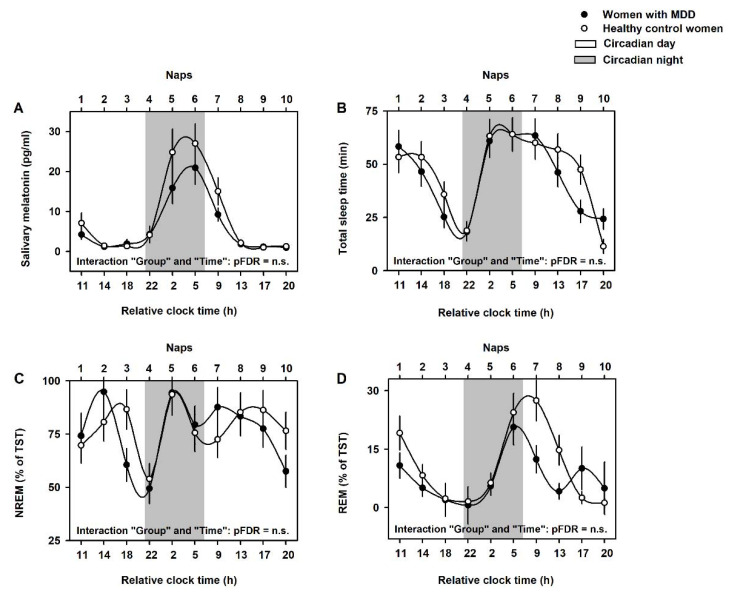
Endogenous circadian melatonin levels and sleep structure during the 40 h multiple nap protocol. We observed no significant interaction effects of “Group” and “Time” for the endogenous melatonin average levels (**A**), total sleep time (TST) (**B**), NREM sleep (**C**), nor for REM sleep (**D**). Data correspond to the mean and standard error of the mean (SEM). Black circles are the average data for women with MDD, and white circles are the average data for the healthy control women. Grey bars correspond to the naps during the circadian night (between ~22:00 h and ~05:00 h).

We assessed whether group differences in dream recall, the number of dreams and dream emotional load were due to differences in the number of naps that occurred during the circadian day/night. We observed no significant differences in the number of naps during the circadian day/night between groups (circadian day naps in healthy control women: 3.6 ± 0.2, circadian night naps in healthy control women: 5.4 ± 0.3; circadian day naps in women with MDD: 3.4 ± 0.3, circadian night naps in women with MDD: 5.6 ± 0.2; *p* = n.s.). Of note, mixed-model analyses of variance of endogenous melatonin average levels indicated significant effects of “Time” (F = 21.2, pFDR < 0.001) and of “Group” (F = 7.8, pFDR = 0.02), but not for the interaction effect of “Group” and “Circadian day/night” (F = 0.8, pFDR = n.s.; Figure 3A).

**Figure 4:**
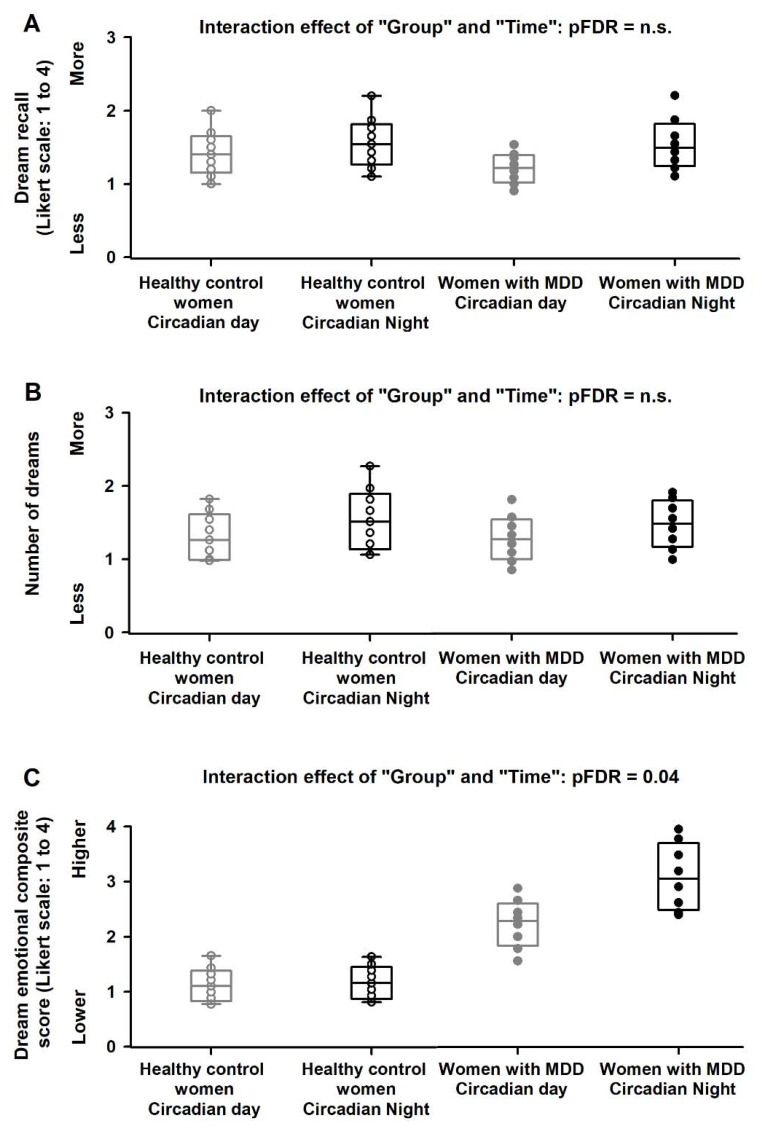
Circadian day/night modulation of dreaming in healthy control women and in women with MDD. We observed no significant interaction effects of “Group” and “Circadian day/night” for dream recall (**A**) and the number of dreams (**B**). In contrast, we observed a significant interaction effect of “Group” and “Circadian day/night” for the dream emotional composite score (**C**). Accordingly, women with MDD had significantly higher (negative) dream emotional composite scores after naps during the circadian night as compared to the healthy control women. Circles are individual data and boxes are the median and the interquartile range.

Mixed-model analyses of variance of dream recall and the number of dreams yielded no significant differences for the effects of “Group”, “Circadian day/night” nor for the interaction effect of “Group” and “Circadian day/night” (all F < 0.9, pFDR = n.s.; Figure 4. In contrast, analyses of the dream emotional composite score yielded significant differences for the effects of “Group” (F = 5.6, pFDR = n.s.), “Circadian day/night” (F = 4.9, pFDR = 0.03) and for the interaction effect of “Group” and “Circadian day/night” (F = 4.2, pFDR = 0.04; Figure 4). Healthy control women did not exhibit significant differences between the circadian day and night (Tukey–Kramer test, *p* = n.s.). Conversely, women with MDD had significantly higher (negative) dream emotional score during the circadian night as compared to the circadian day (Tukey–Kramer test, *p* = 0.009; Figure 4).

### 2.3. NREM/REM Modulation of Dream Recall, Number of Dreams and Dream Emotional Load 

**Figure 5:**
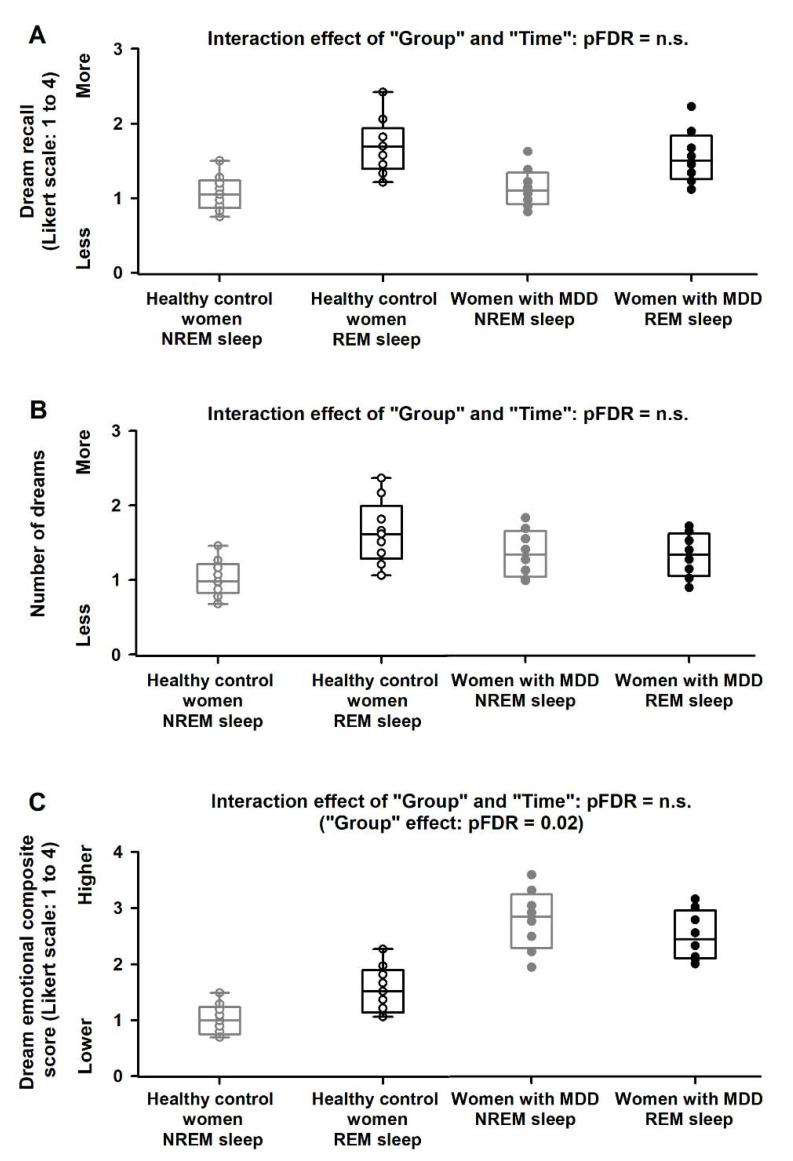
NREM/REM sleep modulation of dreaming in healthy control women and in women with MDD. We observed no significant interaction effects of “Group” and “NREM/REM sleep” for dream recall (**A**) and the number of dreams (**B**). Although we did not observe a significant interaction effect of “Group” and “NREM/REM sleep” for the dream emotional composite score, there was a significant main effect of “Group” (**C**). Accordingly, women with MDD had higher (negative) dream emotional composite score after both NREM and REM naps as compared to the healthy control women. Circles are individual data and boxes are the median and the interquartile range.

We then investigated whether group differences in dream recall, the number of dreams, and the dream emotional load were due to differences in the number of naps that had NREM or REM sleep. We observed no significant group differences in the number of NREM/REM naps (NREM naps in healthy control women: 4.7 ± 0.6, REM naps in healthy control women: 4.0 ± 0.4; NREM naps in women with MDD: 5.6 ± 0.7, REM naps in women with MDD: 3.7 ± 0.9; *p* < 0.1). Mixed-model analyses of variance of total sleep time (TST), NREM and REM sleep yielded no significant differences for the effects of “Group” (all F < 1.2, pFDR = n.s.) nor for the interaction effect of “Group” and “Time” (all F < 1.0, pFDR = n.s., Figure 3B–D). Mixed-model analyses of variance of dream recall and number of dreams yielded significant differences for the effect of “NREM/REM naps” (respectively, F = 8.5, pFDR = 0.001; F = 7.6, pFDR = 0.001). However, there were no significant differences for the effect of “Group” (respectively, F = 1.2, pFDR = 0.3; F = 1.3, pFDR = n.s.) nor for the interaction effect of “Group” and “NREM/REM naps” (respectively, F = 0.8, pFDR = n.s.; F = 0.7, pFDR = n.s.; Figure 5). In contrast, analyses of the dream emotional composite score yielded significant differences for the effects of “NREM/REM naps” (F = 7.6, pFDR = 0.002) and “Group” (F = 5.6, pFDR = 0.02), but not for the interaction effect of “Group” and “NREM/REM naps” (F = 1.2, pFDR = n.s.; Figure 5). Accordingly, women with MDD exhibited significantly higher (negative) dream emotional composite score after both NREM and REM naps, as compared to healthy control women (Tukey–Kramer test, *p* = 0.005; Figure 5).

### 2.4. Association of Dream Emotional Load and Subjective Perception of Mood

**Figure 6:**
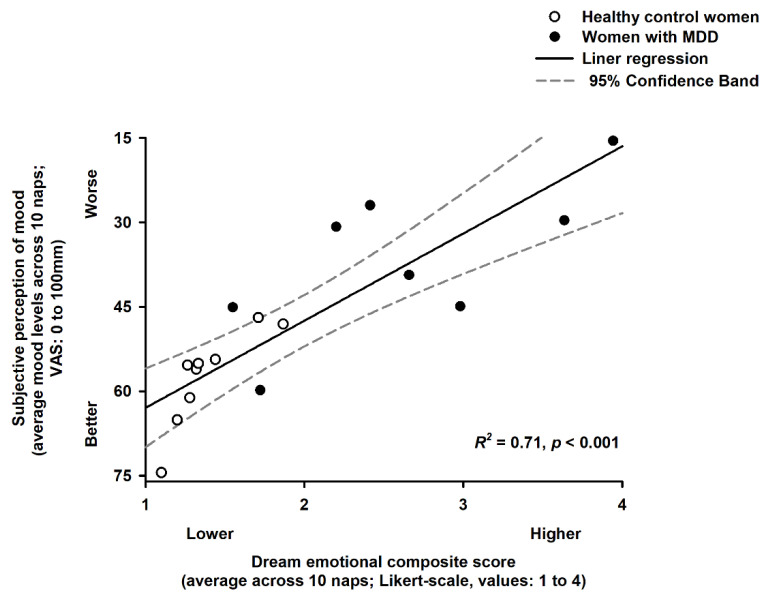
Association of dream emotional load and subjective perception of mood. The dream emotional composite score was significantly associated with the degree of changes in mood levels during the 40 h multiple nap protocol (R^2^ = 0.71; *p* < 0.001). Data averaged across the 10 naps. Black and white circles are, respectively, the individual data for women with MDD and for the healthy control women. Solid and dashed lines correspond to, respectively, the linear regression model and the 95% confidence interval.

Last, we assessed whether there was an association between the magnitudes of dream emotional load with that of subjective perception of mood during the 40 h multiple nap protocol. Accordingly, we observed that higher (negative) dream emotional composite score was significantly associated with worse mood levels in both groups (R^2^ = 0.71; *p* < 0.001; Figure 6).

## 3. Discussion

Our data do not support the two study predictions. First, we anticipated a reduction in dream recall in women with MDD due to an impaired circadian modulation of dreaming. However, we did not observe a dampening or a phase shift of the circadian rhythms of dream recall, of the number of dreams nor of the dream emotional load. Indeed, not only did the young unmedicated women with MDD recall similarly to the healthy control women, they exhibited a more pronounced circadian rhythm of dream emotional load, with a two-fold higher dream emotional composite score after naps during the circadian night. Therefore, our data suggest that young unmedicated women with mild-to-moderate MDD symptoms may not experience a reduction in their ability to recall dreams because underlying circadian processes that foster dreaming are intact.

Individuals with MDD often show circadian rhythm disruption that may underlie the propensity to exhibit severe depressive symptoms [28]. Circadian gene polymorphisms have been associated with mood disorders [29]. Accordingly, alterations of circadian gene networks may influence mood disorders, particularly (moderate-to-severe) MDD, through the disruption of monoamines and dopamine transmission [29]. Furthermore, magnetic resonance imaging (MRI) assessments of the pineal gland (a major site of melatonin synthesis) in current MDD, remitted MDD, bipolar depression and (age and sex-matched) healthy controls indicated smaller pineal volume in individuals experiencing current MDD, which was associated with the severity of their depression symptoms [30]. While that study did not include melatonin assessments, pineal abnormalities may be associated with specific subtypes of MDD and symptom severity. As our MDD group did not include individuals with severe depression, their endogenous circadian rhythms might have remained intact. We previously showed in the same study sample that women with MDD exhibit overall lower melatonin levels as compared to healthy control women, although there was no statistically significant alterations of the amplitude or phase of the endogenous melatonin rhythms [31]. Moreover, we observed that unmedicated women with MDD, despite overall lower mood levels, did not have a disruption of the endogenous circadian mood rhythms (e.g., dampened amplitude or phase advance/delay) [13]. Collectively, women with mild-to-moderate depression may not have altered endogenous circadian rhythms implicated in dreaming, and thus their ability to recall dreams may not be impaired.

Our second prediction was that reduced dream recall in women with MDD would mostly occur after REM naps, as this sleep stage is often associated with higher dream recall and dream emotional load [17,18]. Our data did not support that prediction, as the recall rate and the emotional composite score after REM naps did not significantly differ between groups. In contrast, women with MDD had higher (negative) dream emotional composite score after NREM sleep than the healthy control women. Dreaming was originally thought to occur only during REM sleep; however, growing evidence suggests it also happens during NREM sleep [19,20,21], a sleep stage with a different global EEG signature and neuromodulatory profile (e.g., reduced cholinergic and noradrenergic transmission) [32]. This observation highlights that dreaming is a subjective experience continuing across different sleep stages. Here, we show a higher dream emotional composite score after both NREM and REM in women with MDD, which was not mediated by statistically significant differences between groups for dream recall, the number of dreams or sleep structure (TST, NREM and REM sleep, Figure 3B–D).

Importantly, higher (negative) dream emotional load was associated with worse mood levels during the 40 h multiple nap protocol. This finding may point to a continuum of negative emotions across different behavioural states in depression, whereby (negative) waking thoughts might intrude in dream imagery and vice-versa [33,34,35]. Indeed, individuals who experience unpleasant/hostile moods during the waking day might also experience dreams that are unpleasant and with negative emotions [36]. Collectively, we speculate that these data might help in the potential identification of individuals with depression through their dream emotionality [37].

A critical aspect of our study is that the study groups did not use any medication, as it could have affected sleep and circadian rhythms. Antidepressant intake (e.g., tricyclic antidepressants, selective serotonin reuptake inhibitors) has been shown to lower dream recall rates and enhance positively toned dreams in individuals experiencing primary insomnia [38] and depression [39,40]. The antidepressant effects on altered sleep (e.g., early morning awakening, reduced REM latency) often observed in this patient population [3] might adversely affect the ability to recall dreams and dream emotional salience. However, this assumption requires formal testing in future studies in patients with MDD with and without antidepressant intake.

Strengths to our study include our stringent inclusion/exclusion criteria that ruled out potential confounders, including comorbidities, medication and sleep disruption. Therefore, we could reliably infer that our findings were attributable to circadian/sleep processes underlying major depression and not due to confounders and dissimilarities between groups. Moreover, as we used the Likert-scale Sleep Mentation Questionnaire, we could repeatedly probe dream recall, the number of dreams and the emotional load of dreams, whereas detailed diaries could have influenced dream recall at successive naps. Limitations to our current findings include the relatively low sample size, which therefore requires replication in studies with larger samples. Moreover, we excluded comorbidities, including insomnia, which frequently co-occurs with this depression subtype [41]. Likewise, anxiety is frequently comorbid to disrupted sleep and to MDD [42] and often affects dream recall and its emotional salience [37,43]. Importantly, we only included women in our study. As there are sex differences in dream recall [44] and in human circadian rhythms [45], future studies should include women and men when assessing dream recall and its emotional load in depression.

## 4. Materials and Methods

Other aspects of this study that was designed to test separate, independent hypotheses on sleep, circadian, mood and cognitive performance were previously published [13,31,46,47].

### 4.1. Study Participants

All procedures conformed to the Declaration of Helsinki and the local Ethical Committee (Ethikkommission beider Basel, Basel, Switzerland). Participants provided written informed consent and were recruited via advertisement at different Swiss universities and through online job advertisement pages. In this laboratory protocol, we enrolled eight young women with MDD (age range 20–31 years; age: 24.3 ± 4.5 years, mean ± standard deviation) who fulfilled the complete diagnostic criteria of the Diagnostic and Statistical Manual (DSM-IV) for MDD. All women with MDD were at the onset of MDD and did not have previous episodes of depression. Participants underwent a clinical interview for DSM-IV Axis I Diagnoses of existing symptoms [48] with the same clinical psychologist. Women with MDD enrolled in this study had all of the following symptoms at the time of their clinical interview: “sadness”, “diminished interest or pleasure”, “energy loss”, “reduced feeling of self-worth”, “diminished concentration”, and “social withdrawal”. We also assessed their clinical status using the Structured Interview Guide for the Hamilton Depression Rating Scale with Atypical Depression Supplement (SIGH-ADS) [49]. This consists of the Hamilton–17 item scale (Standard Value ≥ 8; 12.39 ± 2.5) plus atypical items including the Montgomery–Åsberg Depression Scale [50] (MADRS: Standard Value ≥ 13; 16.7 ± 2.1) and the Beck Depression Inventory (BDI: Standard Value ≥ 12); 21.3 ± 6.8) [51]. Two weeks after the study, a follow-up assessment of the BDI (21.8 ± 9.1) confirmed the maintenance of their depressive symptoms. We included only women because they are two-fold more likely to experience MDD as compared to men [31,47]. Furthermore, we included only participants with MDD who were not taking any medication (except for oral contraceptives) and did not have any comorbid medical and sleep disorders; the latter was ruled out by a polysomnographic adaptation night before the laboratory protocol. 

For the healthy control group, we included nine young women (age range 20–31 years; 25 ± 3.3 years), who were not taking any medication (except for oral contraceptives) and did not have any comorbid medical and sleep disorders, as assessed by a full-night polysomnography screening (for details see [47,52]). We included individuals with intermediate chronotypes (morning-evening-type questionnaire rating between 14 and 21 points [41]) to minimize chronotype differences. Age and BMI did not significantly differ between groups (for details, see [47]). Participants were non-smokers, without any drug use, did not perform shift work within the last year before the study, and were required to abstain from intense physical exercise. Participants commenced the laboratory protocol on days 1–5 after menses onset to complete the entire study block within the follicular phase, except for three women with MDD and five women from the healthy control group who were taking oral contraceptives.

### 4.2. Study Design

Participants maintained a regular sleep–wake cycle (sleep- and wake-times within ±30 min of self-selected target time), as verified by wrist activity monitors (Cambridge Neurotechnology^®^, Cambridge, UK) and sleep logs during the week before the study. We calculated each participant’s habitual bedtime by centering the 8 h sleep episodes at their midpoint during the week before the study. The laboratory protocol included a baseline sleep episode in individual rooms devoid of time cues, followed by a 40 h multiple nap protocol, with ten alternating cycles of 150 min of wake/75 min of sleep per nap, followed by one 8 h recovery sleep episode [31] (Figure 1). The baseline and recovery sleep episodes started at each participant’s habitual bedtime. The multiple nap protocol is a validated well-controlled circadian laboratory protocol designed to tease apart the relative contribution of the circadian timing system on a given outcome of interest [31]. This is because such laboratory protocol controls for the masking effects of e.g., changes in light exposure (dim light <8 l× during scheduled wakefulness and 0 l× during scheduled sleep). Moreover, the multiple nap protocol controlled for masking effects of ambient temperature/humidity, posture (i.e., semi-recumbent posture in bed during wakefulness and supine during sleep), time cues, meals (hourly isocaloric meals and water), among others [53]. Because participants completed ten sleep/wake cycles, homeostatic sleep pressure remained low. Thus, the effects of the circadian timing system—without the effects of increased sleep pressure—can be observed on an outcome of interest.

### 4.3. Salivary Melatonin and Classification of Circadian Day and Night

Saliva samples were collected every 30 min during scheduled wakefulness. We used a direct double-antibody radioimmunoassay for the melatonin assays by gas chromatography–mass spectroscopy with an analytical least detectable dose of 0.65 pm/mL; Bühlmann Laboratory, Schönenbuch, Switzerland [54]. To classify whether a nap occurred during the circadian day or night, we determined the mean melatonin levels derived from saliva samples between the upward- and downward-mean crossing points per participant. Accordingly, a nap occurred during the circadian night if the melatonin level of the last saliva sample before the nap was above the individual mean. Otherwise, the nap occurred during the circadian day (for details, see [22]).

### 4.4. Sleep Polysomnographic Measures

Sleep was polysomnographically recorded with the VITAPORT ambulatory system (Vitaport-3 digital recorder, TEMEC Instruments B.V., Kerkrade, The Netherlands). Twelve EEGs channels, two electrooculograms, a submental electromyogram, and an electrocardiogram were recorded. All signals were low-pass filtered at 30 Hz (fourth-order Bessel type anti-aliasing, total 24 dB/Oct) at a time constant of 1.0 s. After online digitization using a 12-bit AD converter (0.15 V/bit) in the range of 610 V and a sampling rate at 128 Hz for the EEG. Sleep stages were scored per 20 s epochs [55]. We expressed NREM and REM sleep as the percentage of total sleep time (per nap) before averaging across participants. A nap was defined as a REM nap if only REM sleep was observed in the last 15 min of a scheduled 75 min nap. A nap was defined as an NREM nap if only NREM sleep was observed in the last 15 min of a given nap. Naps that had only wakefulness in the last 15 min of a scheduled nap were excluded from the data analyses. We used the same nap classification criteria described in [22].

### 4.5. Dream Recall and Dream Emotional Load

We assessed dream recall, the number of dreams and the dream emotional load immediately after each nap (~1–2 min after wake-up time) with the Sleep Mentation Questionnaire (SQM). The latter addresses whether participants recalled their dreams, number of dreams, emotionality, vividness, pleasantness, hostility, and colorfulness [22]. We did not probe the content of the dreams, as this could have influenced dream reports at successive naps. The dream recall questions included: “How much did you dream?” (1: greatly, 2: fairly, 3: relatively little, 4: not at all); “How many different dreams can you remember having?” (none [0], 1, 2, 3, 4, 5, 6, more than 6). Participants had a dream recall if their response to these two questions were not 4 and 0, respectively. To assess dream emotional load, we used the following questions from the Sleep Mentation Questionnaire: “How emotional was your dream?” (Likert-scale from 1 to 4, where 1: greatly and 4: not at all); “How vivid was the dream?” (Likert-scale from 1 to 4, where 1: very vivid and 4: not vivid); “How pleasant was the dream?” (Likert-scale from 1 to 4, where 1: very pleasant and 4: very unpleasant); “How much hostility was in your dream?” (Likert-scale from 1 to 4, where 1: greatly and 4: not at all); “Did you dream in color?” (Likert-scale from 1 to 4, where 1: greatly and 4: not at all). We then constructed a dream emotional composite score with the items emotionality, vividness, pleasantness, hostility and colorfulness. This composite score corresponded to the average of these five emotional components, where higher values reflect a negative emotional load. Participants completed a baseline dream assessment using the SQM within ~1–2 min after wake-up time. Therefore, they had 10 dream assessments within ~1–2 min upon awakening from each nap (Figure 1).

### 4.6. Mood Ratings

Participants had subjective mood assessments every 30-min using the Visual Analogue Scale (VAS) [13]. They were asked to indicate their perception of mood “at the moment” by placing a mark on the VAS that ranged from 0 (“worst ever”) to 100 mm (“best ever”). 

### 4.7. Statistical Analysis

For the analysis, we used the statistical package SAS (SAS Institute Inc., Cary, NC, USA; Version 9.1). We performed mixed-model analyses of variance (PROC Mixed), with between-factor “Group” (healthy controls and women with MDD) and within-factor “Time” (10 naps) and the interaction of “Group” and “Time”. To determine whether group differences in dream recall, the number of dreams and dream emotional load depended on the naps occurring during the circadian day or night, we conducted mixed-model analyses with between-factor “Group”, within-factor “Circadian day/night” and the interaction of “Group” and “Circadian day/night”. Moreover, to assess whether group differences in dream recall, the number of dreams and dream emotional load depended on the naps occurring during NREM or REM sleep, we conducted mixed-model analyses with between-factor “Group”, within-factor “NREM/REM sleep” and the interaction of “Group” and “NREM/REM sleep”. We assessed contrasts with the LSMEANS statement, and P-values were based on the Kenward–Roger corrected degrees of freedom. To control the overall type I error in null hypothesis testing when conducting multiple comparisons, we adjusted the P-values using False Discovery Rates (pFDR values). We report type III tests for fixed effects as it accounts for the effects of covariates, main factors and interactions. Moreover, post-hoc comparisons used the Tukey–Kramer test to account for multiple testing. Significance was set as *p* < 0.05. Lastly, we applied a linear regression model to test whether the average levels of the dream emotional composite score were associated with the average mood levels (derived from the VAS) during the 40 h multiple nap protocol. Data in Figure 2 and Figure 3 correspond to the mean and SEM, and data in Figure 4 and Figure 5 correspond to the median and the interquartile range (i.e., the spread difference between the 75th and 25th percentiles of the data).

## 5. Conclusions

We provide evidence in favour of circadian modulation of dreaming in unmedicated women with MDD, as reflected by intact circadian rhythms of dream recall, the number of dreams and the emotional load of dreams, as well as the circadian day/night differences for the latter. Furthermore, we observed higher dream emotional load in women with MDD irrespective of sleep stage. Lastly, higher (negative) dream emotional load was associated with worsened mood across the 40 h multiple nap protocol. Hence, it is likely that unmedicated individuals with MDD exhibit a continuum of subjective experiences across behavioural states. This holds translational relevance as it might tentatively aid in the identification of individuals at the onset of MDD through their dream emotionality. Ultimately, assessments of dream recall and its emotional load may aid in the understanding of emotional regulation in individuals experiencing depression.

## Data Availability

Data needed to evaluate the conclusions in the paper are present in the paper. We will make the datasets available to other investigators following publication of the final study results, upon reasonable request.
